# Hypoxic TAM-derived exosomal miR-155-5p promotes RCC progression through HuR-dependent IGF1R/AKT/PI3K pathway

**DOI:** 10.1038/s41420-021-00525-w

**Published:** 2021-06-15

**Authors:** Wenyu Gu, Linjing Gong, Xu Wu, Xudong Yao

**Affiliations:** 1 Department of Urology, Shanghai Tenth People’s Hospital, Tongji University School of Medicine, Shanghai, China; 2 Department of Pulmonary Medicine, Zhongshan Hospital, Fudan University, Shanghai, China

**Keywords:** Cancer microenvironment, Renal cell carcinoma

## Abstract

Hypoxic tumor-associated macrophages (TAMs) are related to poor prognosis of patients with clear cell renal cell carcinoma (ccRCC). Exosomes are small lipid-bilayer vesicles that implicated in tumor progression and metastasis. However, whether hypoxic TAM-derived exosomes affect RCC progression within the hypoxic tumor microenvironment has not been elucidated. GSE analysis identified miR-155-5p was upregulated in RCC. Moreover, we quantified levels of miR-155-5p using RT-qPCR, performed immunohistochemical staining in 79 pairs of primary RCC specimens and related them to clinicopathological parameters. Higher miR-155-5p levels were related to more CD163 + TAM infiltration and elevated HIF-1a expression in our cohort. In the in vitro studies, we initially purified and characterized the exosomes from the supernatant of TAMs subjected to normoxia or hypoxia, and then transfected antagomiR-155-5p or control into these TAMs to produce corresponding exosomes. Gain and loss-of-function studies further investigated the effect of transferred hypoxic exosomal miR-155-5p on the cross-talk between TAMs and RCC cells in xenograft model and in vitro co-culture experiments. The results of RNA immunoprecipitation analyses elucidated that miR-155-5p could directly interact with human antigen R (HuR), thus increasing IGF1R mRNA stability. Mechanistically, hypoxic TAM-Exo transferred miR-155-5p promoted RCC progression partially through activating IGF1R/PI3K/AKT cascades. Taken together, transfer of miR-155-5p from hypoxic TAMs by exosomes to renal cancer cells explains the oncogenic manner, in which M2 macrophages confer the malignant phenotype to RCC cells by enhancing HuR-mediated mRNA stability of IGF1R.

## Background

As the most prevalent and aggressive renal cell carcinoma (RCC) subtype, clear cell RCC (ccRCC) is characterized by extreme chemo- and radio-resistance. A well-recognized tumor suppressor gene von Hippel-Lindau (VHL) mutations or loss account for ~75% of sporadic ccRCC [1]. When the cancer progress, oxygen within the necrotic tumor-core becomes depleted, resulting in hypoxia and a higher intracellular level of hypoxia inducible factor (HIF-1α and HIF2α) through degradation of VHL [2]. Accumulating studies show that HIF-1α induction could facilitate the oncogenic effect of hypoxic condition on RCC angiogenesis and tumor growth by activation of AKT and VEGFR kinases [3]. Meanwhile, some studies indicate that reactivation of HIF2α promotes xenograft formation by RCC cell lines lacking VHL via suppressing pVHL’s ability [4]. Besides, the expression levels of HIF2α show positive correlation with increasing signs of cellular atypia, as well as conversion to RCC [5, 6], pointing to an important role for hypoxic microenvironment in RCC.

Tumor microenvironment, orchestrated by intercellular communications, plays a pivotal role in the tumor progression and metastasis [7]. The presence of hypoxia within the tumor microenvironment has been proposed to be the most probable cause of malignant transition. Intratumor oxygen deficiency can alter the secretion of chemokines and other proinflammatory cytokines that mediate the cross-talk between tumor cells and immune cells in the tumor stroma [8]. As the major components of immune cells, resident tumor infiltrating macrophages have been termed as tumor-associated macrophages (TAMs). In particular, TAMs are related to tumor progression and poor survival of patients with RCC [9, 10]. Hypoxic TAMs release macrophage migratory inhibitory factor (MIF), which stabilizes HIF-1α protein, eventually promoting the degradation of basement membranes in tumors and providing an easy escape for tumor cells [11]. In addition, hypoxic regions remote to the blood vessels pose challenges for delivering sufficient amounts of drug to the tumor cells [12]. Hence, manipulation of TAMs can be useful to control the tumor microenvironment.

As mediators of intercellular communication, exosomes are released by various types of cells and generated from multivesicular bodies with 30 to 100 nm in diameter, transferring their cargos known as a wide range of functional mRNAs, miRNAs and proteins [13]. Recently, the intriguing roles of exosomes in tumor microenvironment have gained increasing attention that is reported to be involved in chemoresistance or tumorigenesis [14]. Several studies have described that hypoxia could enhance exosomes release and shedding of pro-angiogenic micro-vesicles [15]. Despite the roles of TAMs in immunomodulation and promotion of oncogenesis are well evident, how TAM-derived exosomes affect RCC progression within the hypoxic tumor microenvironment has not been elucidated.

Exosomes contain rich contents of microRNAs, which post-transcriptionally control the translation and stability of mRNAs [16]. Specifically, exosomal miRNAs secreted by TAMs confer chemotherapy resistance or cancer phenotype to recipient cells [17], indicative of a significant mechanism for cancer initiation and metastasis. Aberrant higher expression of microRNA‐155 (miR‐155) has been found in RCC tissues compared to adjacent normal tissues [18]. To initiate a vicious feed forward cycle between tumor angiogenesis and inflammation, TAMs can also produce inducing miR-155-5p factors, such as IL-1, β-FGF and TNF-a [19]. Moreover, it is widely held that miR-155-5p-expressing cancer cells embody an aggressive malignant phenotype via regulation of angiogenesis, cell migration, invasion, epithelial-to-mesenchymal transition and immune response [20, 21]. We previously demonstrated that miR‐155 was augmented in the renal tissue and HK‐2 cells exposed to hypoxia [22]. It was, therefore, of great interest to investigate the unique role of exosomal miR-155-5p in the cross-talk between macrophages and cancer cells. In the current study, the effect of hypoxic TAM–derived exosomes (TAM-Exo) on RCC progression in vitro and in vivo was explored. We hypothesize that exosomal miR-155-5p derived from TAM under hypoxia was a crucial driver in RCC tumorigenesis. Furthermore, our study will reveal the molecular mechanisms that elicit these functions: miR-155-5p directly shuttled from hypoxic M2 TAMs via exosomes to recipient cancer cells, targeting and binding to human antigen R (HuR), in turn to improve IGF1R mRNA stability and promote RCC carcinogenesis and progression.

## Results

### Characterization of exosomes derived from hypoxic TAMs

**Fig. 1:**
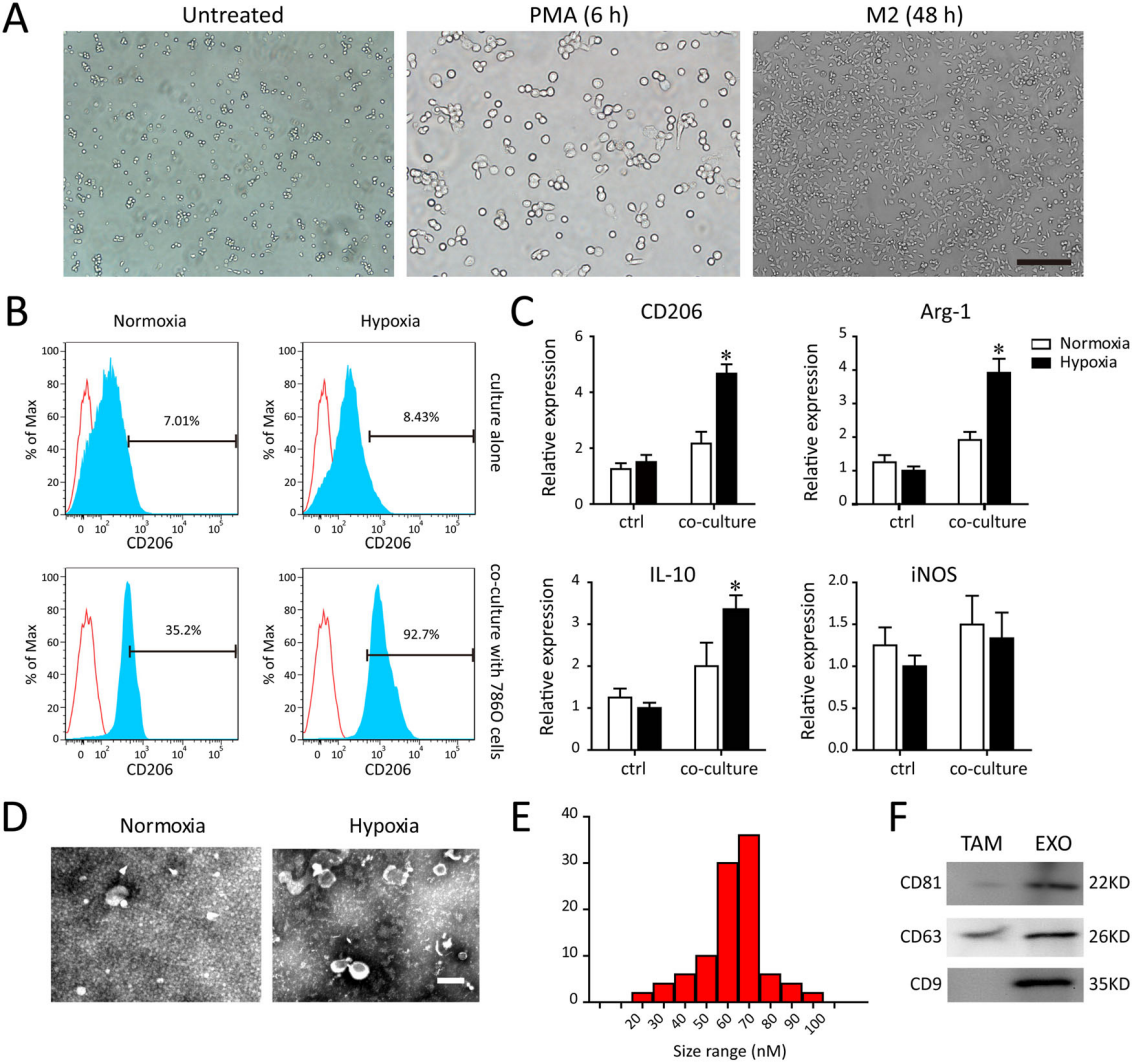
Characterization of TAMs-derived exosomes. **A** After PMA stimulated-differentiation, round and floating THP-1 cells became adherent flattened cells. **B** Flow cytometry analysis for the percentage of M2 polarized macrophages (CD206) after exposing to normoxia or hypoxia and co-culturing with or without 786-0 cells. **C** The RT–PCR detection of typical M2 markers (CD206, Arg1, IL-10, iNOS) in macrophages after exposing to normoxia or hypoxia and co-culturing with or without 786-0 cells. **D** Representative TEM images of TAM-derived exosomes (TAM-exo). Scale bar = 100 nm. **E** Size distributions of exosome fractions isolated from TAM conditioned medium by nanoparticle tracking analysis. **F** Western blot analysis of the exosomal markers (CD81, CD9, and CD63). *P < 0.01 vs. normoxia group.

Human THP-1 monocytes were differentiated into macrophages in the presence of 100 ng/ml phorbol 12-myristate 13-acetate (PMA) for 48 h incubation (data shown as Fig. 1A). Previous study indicated that TAMs infiltrated in renal cancer exhibited an M2 polarized phenotype [9, 23]. To identify the effect of hypoxia on the PMA-induced differentiated macrophages co-cultured with 786-0 cells, the typical M2 markers (CD206) were detected in THP-1. Cytofluorimetry analysis was performed to determine the TAM marker CD206 in macrophages cultured alone (ctrl) or co-cultured with 786-0 cells under normoxia and hypoxia (Fig. 1B). A significant increased percentage of CD206^+^ cells were quantitative identified in macrophages co-cultured with 786-0 cells after exposure to hypoxia, compared with those cultured alone in normoxic conditions. Consistently, a similar phenomenon regarding the M2 polarized phenotype (CD206, Arg1, IL-10) was validated by reverse transcription PCR (RT–PCR) (Fig. 1C). These data illustrate that hypoxic macrophages co-cultured with 786-0 cells have an M2-skewed phenotype, indicative of the predominantly TAMs.

Next, we obtained the TAMs after co-culture with 786-0 cells for 24 h. Moreover, then the exosomes were isolated by ultracentrifugation from the conditioned medium of TAMs subjected to normal or hypoxic culture for an additional 12 h. As showed in Fig. 1D, 1E, the electron micrographs of TEM exhibited the cup‐shape appearance with 30–100 nm in diameter. Exosomes derived from normoxic and hypoxic TAMs showed the difference in the quantity, except for the structure or the fusion capability. The surface markers of hypoxic TAM-exo verified by western blot were abundant in CD81, CD63, and CD9 (Fig. 1F).

### Higher miR-155-5p level correlates with the TAMs infiltration

**Fig. 2:**
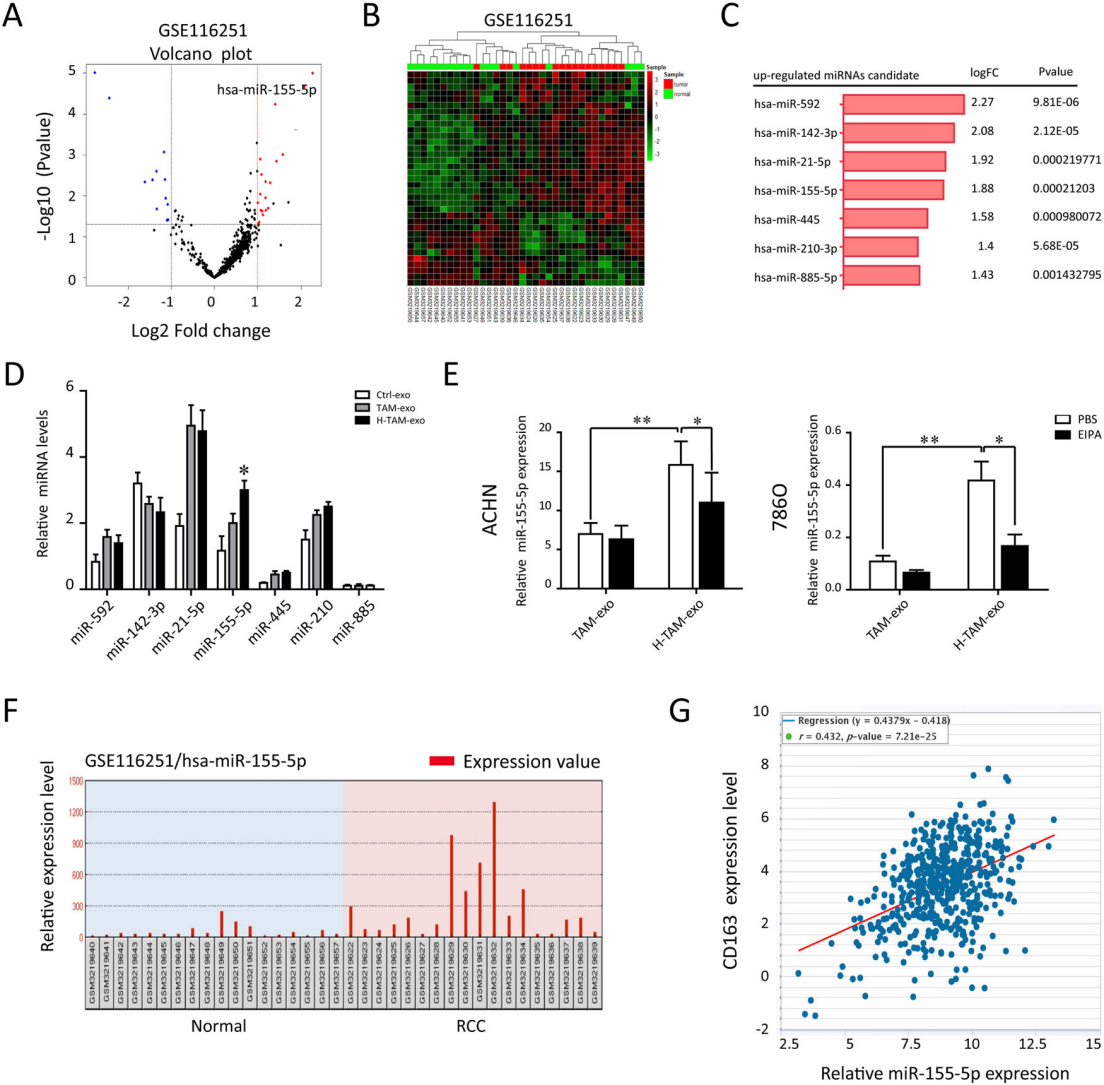
Hypoxia promots miR-155-5p selectively imported into exosomes. **A** The variation in miRNA expression between RCC and normal tissues from GSE116251 was compared. **B** Hierarchical cluster heat map of differentially expressed miRNAs in RCC and corresponding normal tissues from the GSE116251 data set. Red in the heat map denotes upregulation; Green denotes downregulation. **C** The top 7 upregulated miRNAs are listed. **D** The top 7 most abundant miRNAs were measured in exosomes derived from un-activated macrophages (control), TAM and hypoxic TAM (H-TAM). **E** Relative miRNA levels of RCC cells co-cultured with exosome released from TAM or hypoxic TAM. To block the exosome uptake into cells, EIPA (10 μM) was added in the media during co-culture. **F** Data set from GSE11625 showed the enrichment of miR-155-5p in the enrolled RCC individuals. **G** The Pearson correlation analysis of the transcription level of miR-155-5p and CD163 in TCGA database (*P* < 0.001). **P < 0.01, ***P < 0.001.

To determine the expression pattern of miRNAs encapsulated in TAM-exosomes, we first analyzed GSE116251 from the GEO database of RCC tissues. We compared the variation of miRNA expression between RCC and normal tissues via volcano plots (Fig. 2A). In total, miRNAs were upregulated in RCC tissues with fold-changes greater than 2.0. Then, a cluster heap map was used to present the up- and downregulated miRNAs according to the miRNA expression levels (Fig. 2B). Among these, the top 7 upregulated miRNAs were listed in Fig. 2C. To verify the secretion of TAM-Exo, we used qRT–PCR to quantify the exosomal miRNA content in un-activated macrophages (control), normoxic TAM and hypoxic TAM (H-TAM). We focused on miR-155-5p given its abundance, as reflected by the markedly elevated miR-155-5p levels harvested from hypoxic TAM-Exo than those from other groups (Fig. 2D). In addition, we utilized EIPA to disrupt internalization of exosomes into target 786-0 cells. As displayed in Fig. 2E, the relative level of miR-155-5p was significantly increased in 786-0 cells co-cultured with exosomes released from H-TAM compared to cultured alone. Intriguingly, marked downregulation of miR-155-5p was shown in 786-0 cells when pretreated with EIPA, suggesting that miR-155-5p was mainly responsible for this bioactivity. According to the GSE116251, miR-155-5p was significantly upregulated in RCC tissue (*n* = 18) compared to paired normal tissue (*n* = 18, Fig. 2F). To further validate the above data, miR-155-5p levels were positively correlated with TAMs infiltration (CD163^+^ cells) in RCC specimens from TCGA databases (Fig. 2G). These results confirm that hypoxia promote miR-155-5p selectively imported into exosomes.

### miR-155-5p and TAM infiltration associated with the hypoxic tumor microenvironment predict a poor prognosis of RCC patients

**Fig. 3:**
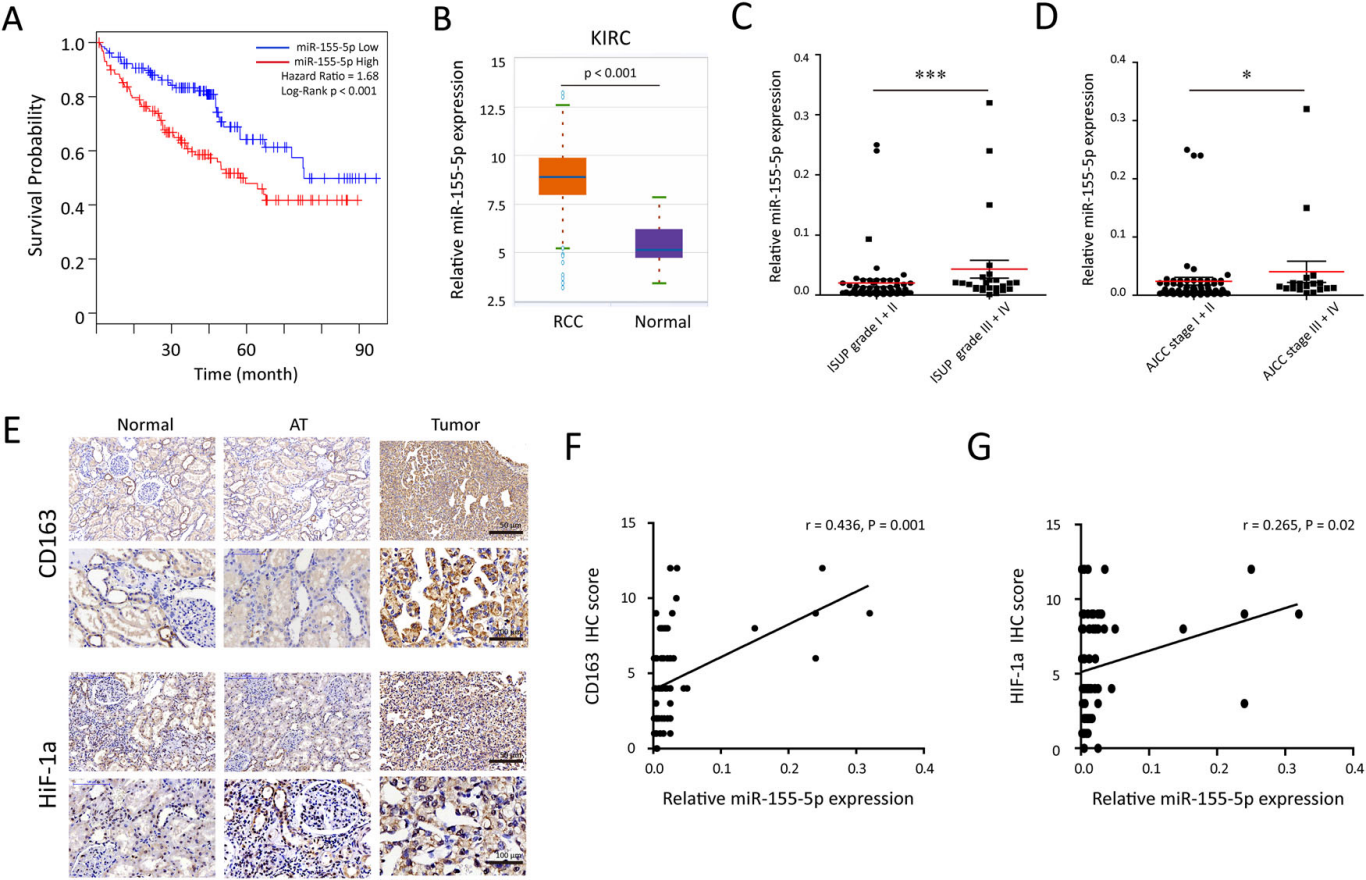
miR-155-5p is correlated with prognosis in RCC patients. **A** A Kaplan–Meier survival analysis for RCC patients according to miR-155-5p expression in the TCGA databases. **B** The expression levels of miR-155-5p in RCC tissues were significantly higher than that in normal kidney tissues (ENCORI, http://starbase.sysu.edu.cn/). **C**, **D** In our 79 pairs of primary RCC cohort, the differences of significant overregulation of miR-155-5p between patients with high or low ISUP grade and AJCC stage were determined by qRT–PCR. **E** Immunohistochemistry analysis of CD163 and HIF-1a. Representative photographs are shown (×200 and ×400 magnifications). **F**, **G** A plot of the relative expression of miR-155-5p vs. HIF-1a and CD163 staining showed their positive correlations in our cohort. Correlation index *r* was calculated using Spearman rank test.

Analysis of TCGA database with >500 human RCC samples showed higher miR-155-5p in RCC predicted a poor prognosis (Fig. 3A). Similar results were observed in renal cancer tissues (*n* = 517) and normal tissues (*n* = 71). The expression levels of miR-155-5p in RCC tissues were significantly higher than that in normal kidney tissues in ENCORI based on TCGA database (Fig. 3B). Next, we performed qRT–qPCR to quantify the levels of miR-155-5p from 79 paired RCC tissues and adjacent tissues in our cohort. As shown in Fig. 3C, D, higher miR-155-5p levels were related to higher ISUP grade (*P* = 0.003) and advanced AJCC stage (*P* = 0.023). To confirm the correlation between miR-155-5p and hypoxic tumor microenvironment, the immunohistochemical staining was applied to examine CD163 and HIF-1a expressions in 79 pairs of primary RCC tissue, adjacent and normal renal tissues (Fig. 3E). The results revealed that more intra-tumoral CD163^+^ cell infiltrations were detected along with higher miR-155-5p expression (*r* = 0.436, *P* = 0.001, Fig. 3F). Importantly, we also noticed the obvious positive correlations between the miR-155-5p levels and the expression of HIF-1a levels in RCC tissues (*r* = 0.265, *P* = 0.02, Fig. 3G). Moreover, we found the expression of CD163 was positively linked to the AJCC tumor stage (*r* = 0.326, *P* = 0.003, data not shown). These data suggest that elevated miR-155-5p expression and TAM infiltration are associated with the hypoxic tumor microenvironment in RCC patients.

### Hypoxic TAM-Exos confer miR-155-5p to recipient cells and modulate their biological functions in vitro

**Fig. 4:**
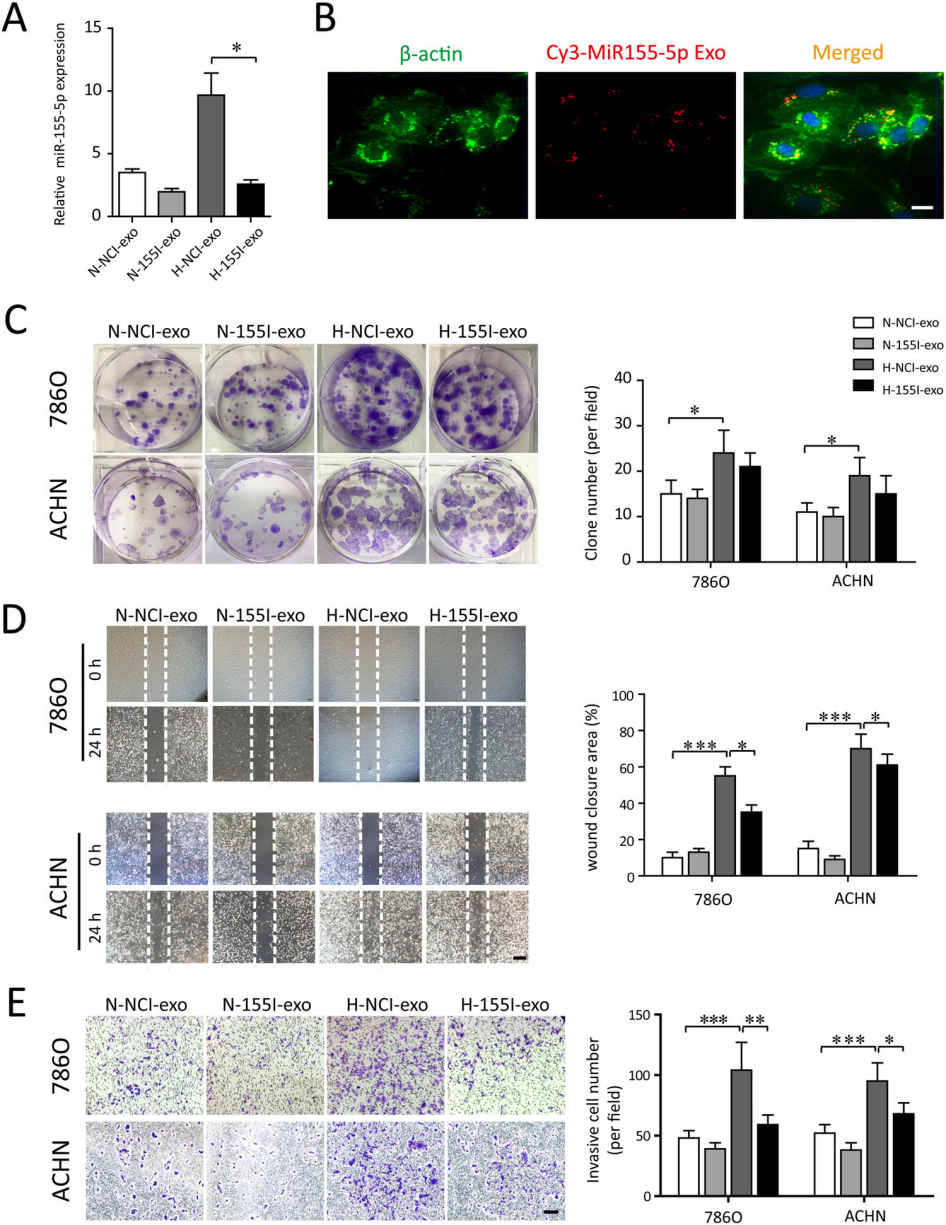
Exosomal miR-155-5p facilitates the proliferation, wound healing and migration of RCC cells in vitro. **A** The normoxic or hypoxic TAMs were transfected with antagomir control and antagomiR-155-5p to produce NCI-exos and 155I-exos. The corresponding exsomal miR-155-5p levels (N-exo+agomir-NC, N-exo+agomiR-155-5p, H-exo+antagomir-NC, H-exo+antagomiR-155-5p) were validated by RT–PCR. **B** The uptake of the Cy3-labeled M2-Exos was observed in RCC cells. **C** 786-0 and ACHN cells were incubated with exosomes derived from the normoxic or hypoxic TAMs, which were transfected with control or agomiR-155-5p, respectively. Clone formation of each group (N-NCI-exo, N-155I-exos, H-NCI-exo, H-155I-exo) were observed and the colony numbers were quantified. **D** The RCC cells were scratched, photographed at time 0, and incubated in the presence of N-NCI-exo, N-155I-exos, H-NCI-exo, H-155I-exo (50 μg/ml). Photographs were taken again after 24 h (Scale bar = 200 μm). Quantification of the closure area was presented as the ratio of closure area to the initial wound area. **E** Representative image of invasion assay. Diagram of invasive cells in response to various exosomes from six random high-power fields (Scale bar = 100 μm) counted in three independent experiments. *P < 0.05, **P < 0.01, ***P < 0.001.

To examine whether hypoxic TAMs modulate their biological functions via transfer of exosomal miR-155-5p onto recipient renal cancer cells, 786-0 and ACHN cells were co-cultured with exosomes released from normoxic TAMs or hypoxic TAMs. We then transfected antagomir control and antagomiR-155-5p into the TAMs to produce NCI-Exos and 155I-Exos. As expected, transfection of TAMs with antagomiR-155-5p resulted in a significant decrease of the exosomal miR-155-5p, in comparison with those transfected with antagomir control (Fig. 4A). The uptake of the Cy3-labeled exosomes was evident in ACHN cells after 12 h of hypoxic incubation (Fig. 4B). These data indicate that miR-155-5p is highly enriched within hypoxia-stimulated TAMs, and can be further transferred to RCC cells via cargoes of exosomes.

The exosomes derived from different sources of TAMs were collected and used in the cellular functional assay and subsequent in vivo experiments. The hypoxic TAM-Exo (H-NCI-exo) substantially promoted colony-formation capacity, whereas the deletion of miR-155-5p exosomes derived from hypoxic TAM (H-155I-exos) significantly abolished this proliferative effect (Fig. 4C). We then conducted wound-healing assay and found that H-NCI-exo resulted in a significant increased cell migration compared with normoxic TAM-Exo (N-NCI-exo). In contrast, H-155I-exos failed to promote a wound-healing effect on the closure area of RCC cells (Fig. 4D). Consistently, enhanced invasion abilities were observed in the H-NCI-exo treated-cell lines, while knockdown of miR-155-5p in hypoxic TAM-Exos disabled the invasive capability of RCC cells (Fig. 4E). Collectively, exosomes delivered from hypoxic TAMs modulate recipient renal cancer cell functions by shuttling miR-155-5p.

### Exosomal miR-155-5p facilitates the malignant phenotype of RCC cells by binding to HuR

**Fig. 5:**
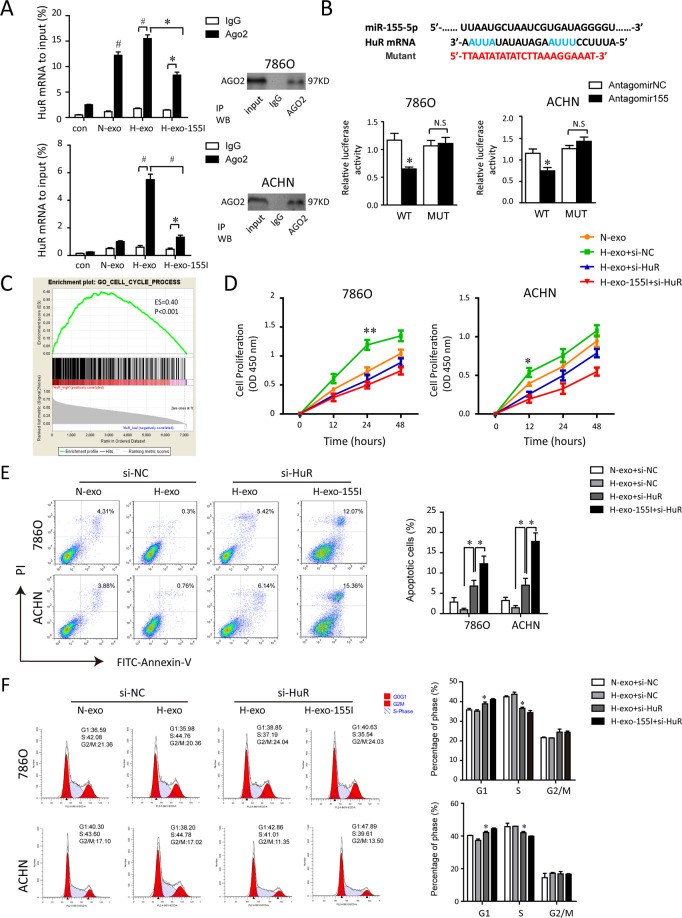
Exosomal miR-155-5p contributes to the malignant phenotypes of RCC cells by binding to HuR. **A** HuR mRNA was associated with amount of exosomal miR-155-5p in Ago2. RNA immunoprecipitation with an anti-Ago2 antibody assessed endogenous Ago2 binding to RNA in RCC cells treated with blank, N-exo, H-exo, H-155I-exo, respectively. IgG served as the negative control. RIP efficiency of Ago2 protein was detected by western blot. The relative HuR mRNA levels were determined by qRT–PCR and presented as fold enrichment in Ago2 relative to input. **B** HuR is a direct target of miR-155-5p. Dual-luciferase reporter assays were conducted to confirm a direct interaction between miR-155-5p and HuR in 786-0 and ACHN cells. Upper panel, sequence alignment of miR-155-5p and its putative-binding sites in HuR mRNA 3′-UTR containing an ARE motifs (AUUA, AUUU). Predicted miR-155-5p target region in HuR (Luc-WT) and mutant type (Luc-MUT) were constructed and transfected into RCC cells with antagomiR-155-5p or control. Luciferase activity is presented as relative luciferase activity normalized to activity of their respective negative control. **C** Results of gene-set enrichment analysis (GSEA) were plotted to visualize the correlation between the expression of HuR and cell cycle gene signatures in TCGA cohort. **D** CCK-8 assays demonstrated HuR silencing inhibited RCC cell growth. **E**, **F** The effect of HuR knockdown on cell apoptosis and cell cycle were analyzed by flow cytometry. The experiment was performed in triplicate. Data are expressed as the mean ± SEM. *P < 0.05, ^#^*P* < 0.01.

To elucidate the putative mechanism by which TAM-Exos and their transferred miR-155-5p regulated RCC cells functions, we found that miR-155-5p contains a binding site for HuR’s mRNA through bioinformatic analysis. Ago2 is an elementary catalytic constituent of RISC involved in RNA cleavage [22, 24, 25]. To investigate the association between miR-155-5p and HuR mRNA, RIP assays were performed on RCC cells with anti-Ago2 antibody beads (Fig. 5A). As expected, miR-155-5p was predominantly enriched in Ago2-containing micro-ribonucleoprotein complexes compared with those harboring control IgG (data not shown), suggesting that the Ago2 protein directly bound to miR-155-5p in RCC cells. Besides, compared to ctrl-IgG immunoprecipitates (IPs), HuR mRNA was preferentially enriched by more than eightfolds in Ago-2 IPs validated by qRT–PCR. In hypoxic TAM-Exo treated 786-0 cells, HuR levels were upregulated in Ago2-IPs compared to normoxic TAM-Exo group, while HuR pulled down by Ago2 antibody were correspondingly reduced relative to the IgG-IPs in miR-155-5p-deleted exosomes group (Fig. 5A).

Then we used dual-luciferase reporter assays to determine the possible interaction between miR-155-5p with HuR in RCC cells. Given the putative-binding sequence for HuR with miR-155-5p, wild-type (Luc-WT) or miRNA-binding site-mutant HuR 3’-UTR-driven luciferase vectors (Luc-MUT) were constructed and transfected into RCC cells with antagomiR-155-5p or control. As shown in Fig. 5B, co-transfecting antagomiR-155-5p notably decreased the firefly luciferase activity of reporter transfected with wild-type plasmid. Conversely, the inhibition was rescued by the mutant construct, indicating HuR was the direct target of miR-155-5p.

GSEA revealed the expression of HuR significantly associated with cell cycle (Fig. 5C). We then employed CKK8 assays to observe cell proliferation rates. HuR siRNA was designed and verified for their interference efficiency. Silence of HuR rescued the increased cell proliferation capacity caused by hypoxic TAM-exosomes. Consistently, H-155I-exos weakened the proliferation promotion effect of hypoxic TAM-exos following HuR knockdown (Fig. 5D). Moreover, flow cytometry analysis demonstrated a significantly increased proportion of apoptosis in si-HuR-transfected RCC cells (Fig. 5E). Additionally, the tumor-promoting effect elicited by transferred hypoxic exosomal miR-155-5p could be further reversed by HuR siRNA. The cell cycle analysis showed that more RCC cells were arrested at G1 phase and less in S phase after silencing exosomal miR-155-5p (Fig. 5F). Altogether, these results indicate exosomal miR-155-5p contributes to the malignant phenotypes of RCC cells through binding to HuR.

### TAM-Exos transferred miR-155-5p enhances HuR-mediated mRNA stability of IGF1R

**Fig. 6:**
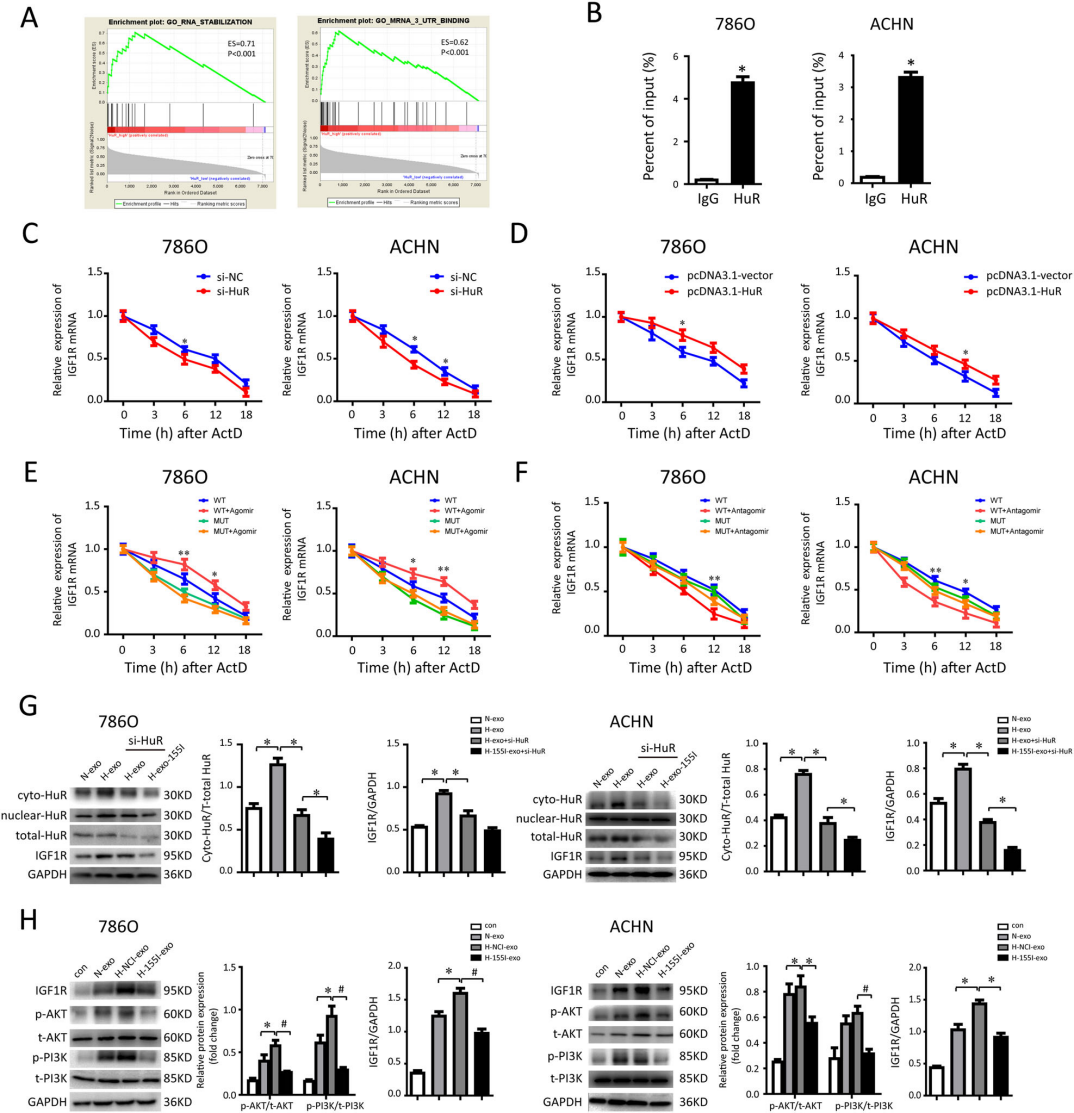
Exosomal miR-155-5p enhances the HuR-mediated IGF1R mRNA stability. **A** GSEA showing HuR expression was positively correlated with mRNA stability and binding in TCGA cohort. **B** RIP assays were performed in RCC cells using HuR antibody to detect IGF1R RNA enrichment in immunoprecipitated complexes. IgG is the negative control. **C** The rate of degradation of the IGF1R mRNA in 786-0 and ACHN cells transfected with control (si-NC) and HuR siRNA over 48 h. Parallel cultures were treated with actinomycin-D and then harvested for RNA extraction at 3, 6, 12, and 18 h thereafter. **D** The rate of degradation of the IGF1R mRNA between the HuR overexpressing (pcDNA3.1-HuR) and control groups using RNA stability assays in RCC cells. **E** The rate of degradation of IGF1R mRNA in the miR-155-5p overexpressing RCC cells transfected with wild-type plasmid vector (WT) or miRNA-binding site-mut vector (MUT). **F** The rate of degradation of IGF1R mRNA in the miR-155-5p knockdown RCC cells transfected with wild-type plasmid vector (WT) or miRNA-binding site-mut vector (MUT). **G** Expressions of HuR and IGF1R protein were detected by western blot after HuR knockdown. Cytoplasmic HuR was normalized to total HuR by Image Lab™ software. **H** Representative photographs of IGF1R, p-PI3K, t-PI3K, p-AKT, and t-AKT protein expressions of 786-0 and ACHN cells collected from each group. GAPDH was used as the loading control. *P < 0.05, ^#^*P* < 0.01.

GSEA analysis in the TCGA identified that high HuR expression was primarily related to the enhanced RNA binding and stability (Fig. 6A). Owing to the sequence complementarity of IGF1R transcript containing a long 5’UTR-binding sites with HuR, we speculated that exosomal miR-155-5p may affect IGF1R expression by regulating HuR-dependent IGF1R mRNA stability in renal cancer. As we hypothesized, RIP assay verified a close binding relationship among HuR and IGF1R mRNA in RCC cells (Fig. 6B). We found that HuR knockdown decreased IGF1R mRNA stability after treatment with actinomycin-D (ActD) to block transcription (Fig. 6C). In line, IGF1R mRNA stability was increased following HuR overexpression (Fig. 6D). Furthermore, to investigate the functional miR-155-5p-HuR binding of seeding AU-rich elements (AREs) sequence on the stability of IGF1R transcripts, angomiR-155-5p or antagomiR-155-5p transfected RCC cells were further treated with ActD, and meanwhile transfected with wild-type 3’-UTR or mutant type, respectively. In wild-type plasmid vector transfected 786-0 cells, the half-life of IGF1R mRNA was ~12 h. In these 786-0 cells, angomiR-155-5p led to the enhanced stabilization of IGF1R mRNA, suggesting a half-life in excess of 12 h (Fig. 6E). In contrast, co-transfection with miRNA-binding site-mutant type and antagomiR-155-5p in 786-0 cells destabilized IGF1R mRNA to the larger extent than the WT group, so that 32% had been degraded before 12 h (Fig. 6F). Overall, these findings indicate that the half-life of IGF1R mRNA is prolonged by functional ARE, partly depending on the interaction between miR-155-5p and HuR.

As an important RNA-binding protein, HuR regulates the mRNA stability via nucleus-to-cytoplasm shuttling. Thus, we examined the effects of exosomal miR-155-5p on HuR distribution. Western blotting showed that after RCC cells incubated with exosomes isolated from hypoxic TAMs, the cytoplasmic distribution of HuR was significantly improved, accompanied with activation of IGF1R. Concomitantly, hypoxic TAM-Exo slightly inhibited IGF1R expression after HuR knockdown. In addition, cytoplasmic the expression of IGF1R was further suppressed despite treatment of H-155I-exos after HuR was silenced (Fig. 6G). The results also confirm that IGF1R is positively modulated by HuR, given that HuR specifically binds to IGF1R mRNA.

It has been suggested that activated IGF axis results in phosphorylation of the PI3K/AKT kinase pathway. To evaluate the effect of exosomal miR-155-5p on PI3K/AKT kinase cascade, similar molecular experiments in RCC cells treated with N-155I-exos or H-155I-exos were performed. As seen in Fig. 6H, hypoxic TAM-Exos significantly enhanced PI3K p85 and AKT phosphorylation. Moreover, silence of miR-155-5p in TAM-Exos failed to activate the IGF1R expression, accompanied with downregulated PI3K/AKT signaling pathway in RCC cells. Taken together, these data suggest that TAM-Exo transferred miR-155-5p mediates the stability of IGF1R mRNA by binding to HuR, thereby facilitating RCC cell proliferation and metastasis.

### Effects of TAMs-derived exosomal miR-155-5p on RCC progression in vivo

**Fig. 7:**
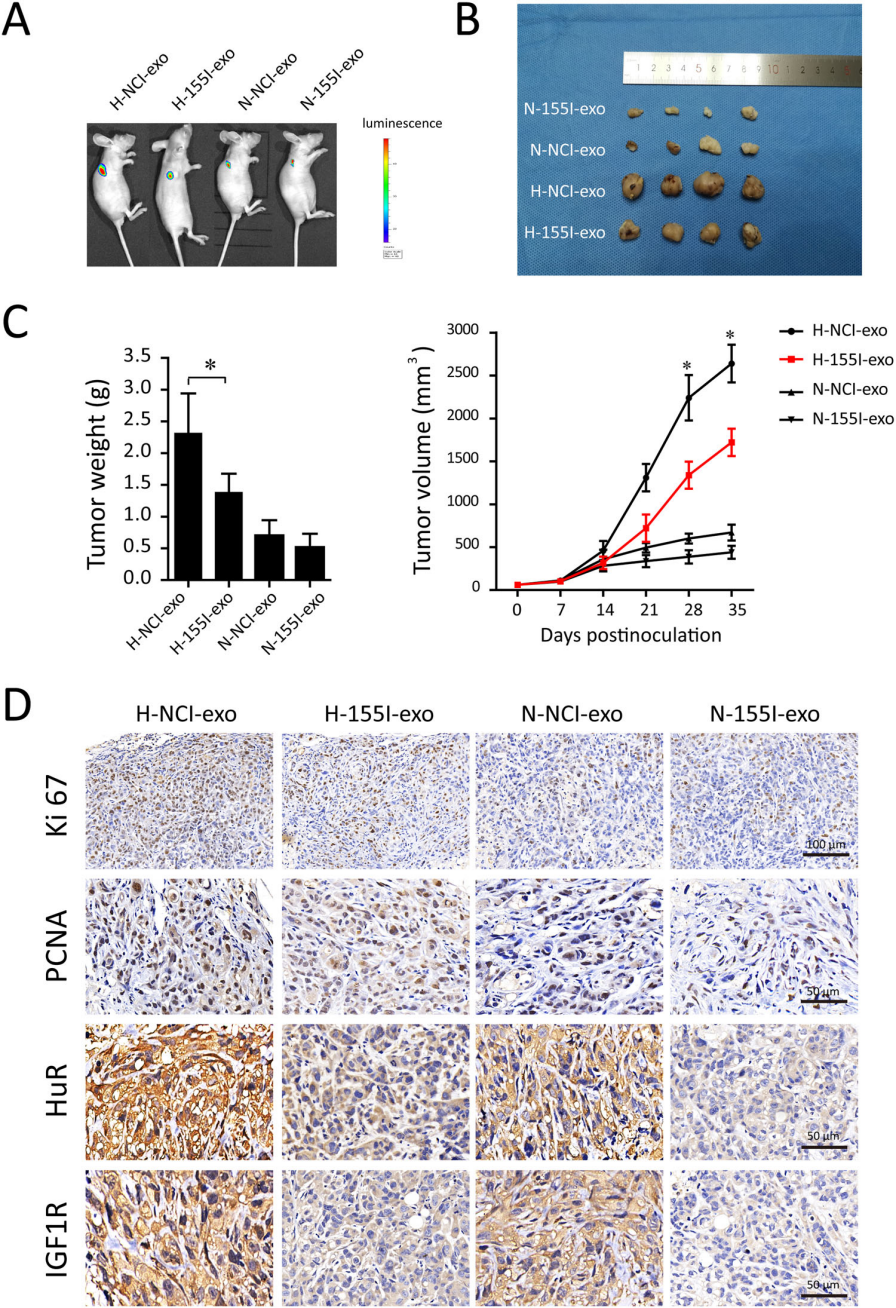
Effects of hypoxic TAM-Exos on RCC progression in vivo. Hypoxic or normoxic TAM-Exos containing miR-155-5p or not were injected into the center of the xenograft tumors to investigate the effects of TAMs-derived exosomal miR-155-5p on ccRCC progression in vivo. N-NCI-exo means normoxic TAM-Exo, N-155I-exo means normoxic TAM-Exo lacking miR-155-5p, H-NCI-exo means hypoxic TAM-Exo and H-155I-exos means hypoxic TAM-Exo lacking miR-155-5p. **A** ACHN cells were subcutaneously injected into BALB/c nude mice. We injected exosomes (10 μg) of different groups into the center of the xenograft tumors twice per week for 3 consecutive weeks (*n* = 6). Presented are representative images (**A**) of orthotopic xenograft animal models viewed by IVIS system in each group on day 35 after tumor cell injection. Representative images (**B**) of the excised tumors from the orthotopic xenograft transplantation. **C** Growth curves and tumor weight of subcutaneous xenograft tumors. **D** The immunohistochemistry analyses for Ki67, PCNA, HuR and IGF1R staining were carried out on ACHN cells xenograft tumor sections. Representative pictures were shown. *P < 0.01.

To investigate the effect of TAMs-derived exosomal miR-155-5p on tumor growth, hypoxic or normoxic TAM-Exos containing miR-155-5p or not were then injected into the center of the xenograft tumors. Xenografts injected with hypoxic TAM-Exos grew statistically significantly larger compared with those treated with normoxic TAM-Exos. Importantly, it was found that miR-155-5p-deleted exosomes abrogated the hypoxic TAM-Exos mediated tumor progression in mice (Fig. 7A, B). Consistent with these results, the tumors of mice in the normoxic TAM-Exos group were decreased in size and volume compared to those in the hypoxic TAM-Exos group (Fig. 7C). Multiple studies revealed dense cytoplasmic distribution of HuR was associated with poor RCC-specific survival [26]. Besides, the IGF-1 and its receptors have crucial roles in RCC tumorigenesis. In vivo experiments confirmed that hypoxic TAM-Exos activated HuR and IGF1R expressions, as indicated by the IHC staining. Meanwhile, immumohistochemical staining of the tumor tissues showed that miR-155-5p-deleted exosomes decreased the expression level of PCNA and Ki67 compared to the NC groups, indicative of lower capabilities of tumor growth (Fig. 7D). Taken together, these data demonstrate that hypoxic TAM-derived exosomal miR-155-5p can promote RCC progression through HuR/IGF1R axis in vivo.

## Discussion

It is widely accepted that extensive TAM infiltration into the RCC microenvironment can promote proliferation, invasion, and immune dysfunction [27]. In this study, we initially purified exosomes from the supernatant of TAMs, and explored the expressive profiles of exosomal miRNAs derived from hypoxic and normoxic TAMs using qRT–PCR. Exosomes derived from hypoxic TAMs could shuttle the increased levels of miR-155-5p, which render the recipient cells in the normoxic region more invasive and aggressive. Furthermore, we observed the high expressions of CD163 and HIF-1a in clinical specimens of RCC. High CD163 + TAM infiltration predicted a poor overall survival of RCC patients. Finally, we demonstrated that significant upregulated miR-155-5p levels in renal cancer tissues were closely related to the advanced TNM stage and hypoxic status of tumors. Our finding shows that transfer of exosomes from hypoxic M2 TAMs to renal cancer cells explains the manner in which immune cells are involved into tumor progression.

Our data also provide biological rationales for CD163 + TAMs as a prognostic biomarker and potential therapeutic intervention target for RCC. Here, we found that TAMs adapt to the hypoxia status were primarily macrophage subpopulation with M2 phenotype. Numerous studies have pointed out that RCC-related immune dysfunction was attributed to anti-inflammatory cytokines, such as IL-10 produced by M2 macrophage [28]. In addition, the importance of HIF-1a in RCC is indicated by several reports demonstrating that positive immunohistochemical staining of HIF-1a correlates with higher grade tumor and poor prognosis [29]. Actually, the biological activities and molecular mechanisms of TAM-released exosomes communicated with renal cancer cells under hypoxia microenvironment have not been well clarified. To our knowledge, it is first to document that exosomes function as a bridge between hypoxic TAMs and renal cancer cells by transferring miRNAs. A growing body of evidence show that hypoxia-induced TAM enrichment and M2 polarization [30]. The exosomes derived from hypoxic regions drive cells toward a pro-tumorigenic phenotype [31]. We suggest that the hypoxic microenvironment stimulates TAMs to produce exosomes that carry increased amounts of miR-155-5p.

Previous studies have shown that hypoxia and VEGF induced miR-155-5p expression in a HIF-1a-dependent manner [32]. Regarded as an “OncomiR,” miR-155-5p shows features of oncogenicity by targeting SOCS1, FOXO3 in multiple types of cancer cells [33]. In accordance with our aforementioned observations, miR-155-5p from M2 macrophage–derived exosomes were transferred to colorectal cancer cells and associated with the malignant progression [34]. Similarly, Kong et al. demonstrated that miR-155-5p downregulated VHL expression to promote tumor angiogenesis, late-stage, and lymph node metastasis in breast cancer [35]. However, it has been reported that miR-155-5p^−/−^ mice or knockdown showed increased tumor growth and impaired the ability of these cells to mount a proinflammatory response [36]. As miR-155-5p bridged the link between inflammation and cancer, interestingly, miR-155-5p overexpressing TAMs were shown to be repolarized from M2 to M1 ex vivo [37]. However, some investigators reported that macrophages deficiency in miR-155-5p appeared to skew toward the alterative M2 phenotype, which recapitulated the alleviated colitis feature of miR-155-5p^−/−^ mice [38]. Collectively, this evidence highlights the central role of alternative M2 skewing for miR-155-5p function probably through alternative mechanisms. Given the different cell types, stimuli, and tumor models used, different experimental results regarding the role of miR-155-5p in the polarization of TAMs are controversial.

In this article, we showed that these tumorigenic effects elicited by miR-155-5p-rich exosomes were largely dependent on miR-155-5p, which in turn increased the cytoplasmic levels of HuR protein to upregulate IGF1R signaling in target cells. Of note, it should take responsibility for a remarkable upregulation of HuR. Recently, Al-Haidari and colleagues revealed the role of miR-155-5p-dependent regulation of HuR expression in colon cancer cells migration [39]. In contrast, Stefanie implicated the pivotal role of HuR in enhancing VHL-mediated p53 translation in VHL-expressing RCC cells [40]. They also found that the markedly abundant cytoplasmic HuR contributed to VHL + tumor suppressive functions in renal cell carcinoma.

It has been well-recognized that RNA-binding protein HuR bound the 3′UTR of the mRNA, which bears U- and AU-rich sequences [41]. In this way, HuR is involved in regulating the stability of mRNAs, such as VEGF mRNA, by binding to AREs in the mRNA 3′ UTR [41]. RNA IP and western blot analysis revealed that HuR directly interacted with miR-155-5p, and silencing of HuR effectively eliminated the effect of exosomal miR-155-5p on IGF1R expression in RCC cells. This finding indicates that exosomal miR-155-5p requires HuR to maintain EGFR mRNA stability. Consistent with our results in cultured cells, another study demonstrated that induction of the tumorigenic effect of miR-155-5p in hepatocellular carcinoma led to the upregulation of IGF-II and IGF-IR downstream signaling cascades [42].

Wu et al. [43] has convincingly showed that HuR plays a fundamental role in the process of tumorigenesis via stabilization of key mRNAs. IGF1R pathway is part of the insulin-related signaling network that crucially implicated in disease progression [44]. IGF1R mRNA also contains some AREs, which therefore indicates the possibility of IGF1R binding with UTR [45]. Through modulating mRNA stability, it is more effective to allow rapid adaptation and maximum cell survival than de novo proteins production in cancer cells. Given the known role of the IGF axis in cell interactions/proliferation, ECM attachment and metastatic spread, it is highly likely that the upregulated IGF1R signaling resulted in high cellular invasiveness, indicative of a poor prognosis even in low-stage of RCC [42, 46]. Notably, a recent report showed that IGF-IR overexpression promoted tumor growth and metastasis in mice model [47]. In agreement with this, we observed upon the hypoxic TAM-exosomes stimulation, recipient renal cancer cells exhibited upregulated HuR-dependent IGF-IR signaling, thus leading to the activation of PI3K/AKT downstream pathway. Considering the variations among different types of tumors, the tissue specific molecular mechanisms of miRNAs that elicit these functions in response to hypoxia warrant an in-depth investigation.

**Fig. 8:**
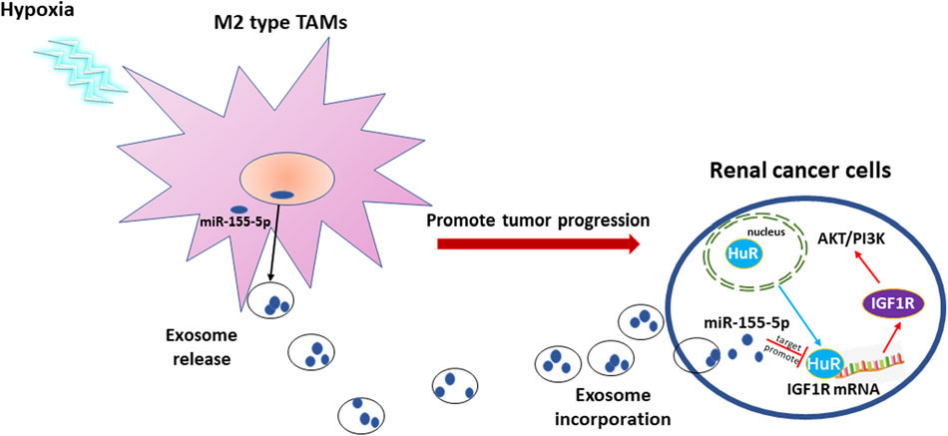
The schematic diagram to illustrate hypoxic TAM-derived exosomal miR-155-5p promotes RCC progression. The schematic diagram shows the mechanism that transfer of miR-155-5p from hypoxic TAMs by exosomes confer the malignant phenotype to RCC cells by enhancing HuR-mediated mRNA stability of IGF1R.

Our study demonstrates that miR-155-5p is closely related to the advanced TNM stage that predicts a poor prognosis of RCC patients. Taken together, hypoxic exosomal miR-155-5p derived from M2 TAMs confers the malignant phenotype to RCC cells by enhancing HuR-mediated mRNA stability of IGF1R (Fig. 8). Along with previous reports, our results suggest that as the messengers that transport signals between cells, exosomes are important components of the hypoxic tumor microenvironment. Preventing exosomal transfer has emerged as an attractive drug target for cancer therapy.

## Materials and methods

### GEO data

MiRNA filtration was carried out on Gene Expression Omnibus (GEO) database (http://www.ncbi.nlm.nih.gov/geo/, GEO accession: GSE116251). There were a total of 18 paired tumor and adjacent normal tissues included in this RCC expression data set. The microRNA expression profile was firstly calibrated and standardized using the R package, and then differentially expressed microRNAs (DE miRNAs) were identified using the limma package with *P* < 0.05 and |log2FC | > 1.

### Surgical specimens

Matched RCC tissues and their corresponding normal or adjacent tissues (*n* = 79) were obtained from patients in the Department of Urology, Shanghai Tenth People’s Hospital, immediately snap frozen in liquid nitrogen, and stored at −80 °C for RNA and protein extraction. Informed consent was obtained from the patients, and the research procedure was approved by the Medical Ethics Committee of Shanghai Tenth People’s Hospital. All included RCC cases were clinically and pathologically identified to be the clear cell type. Clinical data including stage (AJCC, 2002 version) and grade (ISUP grade I to IV) were assembled in a computerized database for each participant. The results published here are in whole or in part based upon data generated by the TCGA Research Network: http://cancergenome.nih.gov/.

### Cell culture and transfection

Human monocytic THP-1 cell line (Cell Bank of the Chinese Academy of Sciences, Shanghai, China) was cultured in Roswell Park Memorial Institute (RPMI 1640, Gibco, USA) culture medium containing 10% fetal bovine serum. Human RCC cell lines 786-0 and ACHN were purchased from American Type Culture Collection (ATCC) (Manassas, VA, USA).

THP-1 monocytes were differentiated into macrophages with 100 ng/ml phorbol 12-myristate 13-acetate (PMA, Sigma, P8139) for 48 h. We first obtained the TAMs after THP-1 co-cultured with 786-0 cells for 24 h. The antagomir-155-5p, agomiR-155-5p and their corresponding controls were synthesized by the Ribobio Company (RiboBio, Guangzhou, China). In some experiments, TAMs were transfected with antagomiR-155-5p (25 nM) or antagomiR control for 48 h using Lipofectamine 2000 in Opti-MEM reduced serum media before collection of exosomes from the cell media. For hypoxia treatment, TAMs were cultured under 1% O_2_ conditions, balanced with N_2_ in an O_2_/CO_2_ incubator (Sanyo) during transfection. The control group was treated in 20% O_2_ (normoxic) conditions. After 24 h hypoxia or normoxia culture, transfected TAMs were washed with PBS and the culture medium were harvested 2 days later. Subsequently, these exosomes were processed appropriately for the different assays. For experiments involving co-culture of exosomes and 786-0 cells, cells were treated with or without EIPA (10 μM, Sigma, Oakville, ON, Canada).

For the mRNA stability assays, the PcDNA3.1-HuR, HuR small interfering RNA (HuR siRNA) or control plasmids were transfected into RCC cells using Lipofectamine 2000 (Invitrogen) according to the manufacturer’s instructions. After 48 h of transfection, cells were used for further experiments.

### Flow cytometry

For detection of CD206-positive M2-macrophges, the control and experimental cells were fixed with 3.7% paraformaldehyde for 20 min at 37 °C, washed with PBS twice and then suspended in PBS and incubated with FITC conjugated anti-CD206 antibody for 1 h at 4 °C. Finally, 10,000 viable cells were harvested and further analyzed using FACS flow cytometer (BD Biosciences, USA). For cell cycle analysis, cells were harvested and fixed in pre-cold 70% ethanol at 4 °C overnight, then stained with propidium iodide (PI). In cell apoptosis experiments, treated cells were harvested and stained with Annexin V/propidium iodide for 30 min in the dark. After washing with PBS, cells were finally analyzed by flow cytometry (Becon Dickinson FACS Calibur, NY, USA).

### Isolation of exosomes and identification

Exosomes were collected from TAM medium after ultracentrifugation as described previously [16]. Firstly, the conditioned medium from TAMs was centrifuged at 3000 × *g* for 15 min, and at 12,000 × *g* for 30 min at 4 °C to remove cells and debris. Exosomes were purified after ultracentrifugation. Then, the isolated exosomes were resuspended with (i) 2% glutaraldehyde in 0.1 mol/L phosphate buffer, and spotted on carbon coated grids (200 mesh; Canemco, Lake field, ON, Canada) for transmission electron microscopy (TEM); (ii) radio immunoprecipitation assay (RIPA) buffer for Western blot or Trizol for total exosomal RNA; (iii) PBS for cell treatment or functional assay in vitro. Western blot analysis was performed to evaluate the expression of CD9, CD63 and CD81. Sizing diameters of exosomes was quantified by nanoparticle tracking analysis (NTA) with a Nanosight LM10 instrument (NanoSight Limited, Amesbury, UK), as described. For exosome uptake experiments, Cy3-labeled miR-155-5p mimics were transfected using Lipofectamine 3000 (Life Technologies), according to the manufacturer’s instructions. Next, exosomes were incubated with RCC cells. Photomicrographs were captured under a confocal microscope (Nikon, Japan).

### Colony formation assay

RCC cells were seeded into six well plate (2 × 10^3^ per well). Then, the cells were exposed to hypoxic TAM-Exo, TAM-Exo or miR-155-5p-deleted exosomes (50 μg/ml). After 24 h, cells were changed into fresh medium containing 10% FBS and incubated in a 37 °C, 5% CO_2_ incubator for 14 days until cells grew into macroscopic colonies. Finally, the medium was removed, and the colonies were stained by 0.1% crystal violet and counted under an optical microscope (Nikon, Tokyo, Japan).

### Wound-healing assay

RCC cells were plated in 6-well plates (2 × 10^5^ cells/well) and wounded by scratching with a 200 μl pipette tip, subsequently, washed with PBS to remove the debris and smooth the edge of the scratch. The serum-free EBM-2 medium (1000 μl) with exosomes from different sources (50 μg/ml) was added to the well. Cells were photographed immediately (0 h) and 24 h later. The level of migration was measured by the ratio of residual closure area of wound (An) to initial wound area (A0) as follows: migration area (%) = (A0 – An)/A0 × 100.

### Invasion assay

Briefly, 3 × 10^5^ RCC cells were seeded on the upper surface of Matrigel-coated membrane inserts (BD Biosciences, San Jose, USA) in serum-free medium, and the lower chamber contained medium with exosomes from different sources. After 24 h of incubation, cells that had migrated from the upper part of the filter to the lower part were fixed with 4% paraformaldehyde and stained with 0.5% crystal violet for 30 min. The invasive cells were counted and photographed in three random views.

### Cell proliferation assay

Cells were seeded into 96-well culture plates at a density of 2 × 10^3^ cells per well and cultured in serum-free medium containing 50 μg/ml hypoxic TAM-Exo, TAM-Exo or miR-155-5p-deleted exosome or an equal volume of PBS. At 0, 6, 12, 24, and 48 h, a Cell Counting Kit-8 assay (CCK-8; Dojindo) was used to assess cell proliferation (10 μl per well) according to the manufacturer’s instructions. Data were collected from three replicates.

### Quantitative reverse transcription PCR (qRT–PCR)

qRT–PCR for analysis of mRNA and miRNA expression was performed as previously described [24]. The primers were synthesized by RiboBio (Guangzhou, China). The expression of indicated genes (CD206, Arg1, IL-10, iNOS, HuR) was normalized to GAPDH using 2^−∆∆CT^ method. Specially, the primers were as follows: hsa-miR-155-5p specific sense 5′-GGGTTAATGCTAATCGTGATAGGGGT-3′. U6 antisense; 5’-CGAATTTGCGTGTCATCCTTG-3’. Here, miR-155-5p expression was normalized to U6 snRNA (ΔCt = Ct miR-155-5p-Ct U6). For IGF1R mRNA stability assays, the transfected RCC cells were treated with 5 mg/ml Actinomycin-D (Sigma) for the indicated times before total RNA was extracted and then performed the qRT–PCR.

### Western blot

For nuclear and cytoplasmic protein extraction, samples were subjected to subcellular fractionation using the extraction kit (Biotech, Nanjing, China). The protein concentration of the lysates was determined separately by stripping the membranes. Western blot was performed as previously described [16]. In brief, the protein samples were lysed and electrophoresed by using 10% SDS gels, and then transferred onto polyvinylidene difluoride (PVDF). Then, the membranes were incubated with 5% skim milk and following primary antibodies overnight at 4 °C: CD63 (1:1000, Cell Signaling, USA), CD9 (1:1000, Proteintech, USA), CD81 (1:1000, Proteintech), HuR, phospho-Akt, AKT, phospho-PI3K, total PI3K and IGF1R (1:1000, CST, Boston, USA). Subsequently, horseradish peroxidase (HRP)-conjugated anti-rabbit or anti-mouse IgG (1:2000, ZSGB-BIO, USA) secondary antibody was added at room temperature for 1 h. The target proteins were visualized using the ECL (EMD Millipore). Densitometric analysis for the quantification of the bands was performed using Quantity One software (Bio-Rad Laboratories, USA).

### RNA immunoprecipitation

We harvested and lysed RCC cells for RNA immunoprecipitation (RIP) experiments. The extract was then incubated with RIP buffer containing magnetic beads conjugated with human Ago2, HuR antibody or negative control IgG (Millipore, USA). An aliquot of lysate was removed as an input control. The purified RNA was determined by qRT–PCR and normalized to the input control.

### Luciferase activity assay

The wild-type or mutant 3’UTR regions of HuR, containing the miR-155-5p-binding sites, were synthesized by Shanghai Heyuan Biotechnology. RCC cells with miR-155-5p knockdown or negative control were transfected with WT-HuR-3′-UTR and MUT-HuR-3′-UTR. Cells were collected 48 h after transfection, and the relative luciferase activity was measured by the Dual-Luciferase Assay System (Promega, Madison, WI, USA) and expressed as Firefly (F)/Renilla (R).

### Gene-set enrichment analysis

Gene-set enrichment analysis (GSEA) was used to explore pathways and gene sets associated with HuR. Gene expression profiles of 530 renal cancer samples were downloaded from TCGA-KIRC data set. According to the HuR expression order, the samples were divided into high expression group (*n* = 265) and low-expression group (*n* = 265), respectively. GSEA v3.0 was used to determine whether the members of the gene sets from the MSigDB database were randomly distributed at the top or bottom of the ranking.

### Histological examination

Briefly, tumors were fixed, embedded and incised to 4 μm thick as previously described [16]. Slides were incubated with primary antibodies with indicated dilution ratio at 4 °C overnight. Then slices were incubated in biotinylated goat anti-rabbit IgG antibody (Invitrogen) at room temperature for 1 h, followed by staining with DAB Kit (Origene, China). The immunohistochemical staining of human RCC cancer tissues and their corresponding normal tissues were also performed. Three fields were chosen from each slide randomly and quantitatively determined on high-power (*100) microscopic fields (HPF). The immunostaining score was calculated by multiplying the intensity (0 = negative, 1 = canary yellow, 2 = claybank, 3 = brown) and the positive cell percentage scores (1 = <25%, 2 = 25% to 50%, 3 = 51% to 75%, 4 = >75%).

### In vivo animal experiment

ACHN cells (6 × 10^6^ in 0.1 ml of sterilized saline) were injected subcutaneously into the right side of 4–5-week-old male BALB/c nude mice to generate potential tumors for 2 weeks. Subsequently, mice were randomly divided into four groups (six mice per group). Equal numbers of exosomes (10 μg) from different sources were injected into the center of the xenograft tumors twice per week for 3 consecutive weeks. Tumor development was monitored by the Fluorescent Imager. Tumor volume was measured in three dimensions (a, b, c) at the indicated time points using calipers and calculated as abc × 0.52. Animals were sacrificed 3 weeks post implantation of exosomes. Tumor weight was determined after mice sacrifice. Tumors were dissected and the section was fixed in formalin and embedded in paraffin for IHC. Researchers are blinded to the grouping information.

### Statistical analysis

Combined with the clinical information in TCGA-KIRC database, the standard Kaplan–Meier univariate curve analysis was adopted via the R software survival package to identify the prognosis of miR-155-5p. It was considered miR-155-5p had potential prognosis value when *P* < 0.05. For in vitro and in vivo analysis, all data are presented as the means ± standard errors (SEM) from at least three independent experiments. Individual data in experimental groups were compared using the two-tailed Student *t*-test. Comparison among groups was performed by one-way ANOVA with Bonferroni post hoc. *P* < 0.05 was considered statistically significant. Statistical analysis was performed using GraphPad Prism 7 (GraphPad, USA) and SPSS 20.0 (IBM Corporation, USA).

## Data Availability

The data used to support the findings of this study are available from the corresponding author upon request.
